# The Association between Atrium Electromechanical Interval and Pericardial Fat

**DOI:** 10.1371/journal.pone.0097472

**Published:** 2014-05-19

**Authors:** Tze-Fan Chao, Yau-Huei Lai, Chun-Ho Yun, Chih-Hsuan Yen, Kang-Ling Wang, Yenn-Jiang Lin, Shih-Lin Chang, Li-Wei Lo, Yu-Feng Hu, Chung-Lieh Hung, Jen-Yuan Kuo, Hung-I Yeh, Shih-Ann Chen

**Affiliations:** 1 Division of Cardiology, Department of Medicine, Taipei Veterans General Hospital, Taipei, Taiwan; 2 Institute of Clinical Medicine, and Cardiovascular Research Center, National Yang-Ming University, Taipei, Taiwan; 3 Division of Cardiology, Department of Internal Medicine, Mackay Memorial Hospital, Taipei, Taiwan; 4 Department of Medicine, Mackay Medical College, and Mackay Medicine Nursing and Management College, Taipei, Taiwan; 5 Division of Radiology, Department of Internal Medicine, Mackay Memorial Hospital, Taipei, Taiwan; Emory University, United States of America

## Abstract

**Objectives:**

Pericardial fat (PCF) may induce local inflammation and subsequent structural remodeling of the left atrium (LA). However, the adverse effects of PCF on LA are difficult to be evaluated and quantified. The atrial electromechanical interval determined by transthoracic echocardiogram was shown to be a convenient parameter which can reflect the process of LA remodeling. The goal of the present study was to investigate the association between the electromechanical interval and PCF.

**Methods and Results:**

A total of 337 patients with mean age of 51.9±9.0 years were enrolled. The electromechanical interval (PA-PDI) defined as the time interval from the initiation of the P wave deflection to the peak of the mitral inflow A wave on the pulse wave Doppler imaging was measured for every patient. The amount of PCF was determined by multi-detector computed tomography. The PA-PDI interval was significantly correlated with the amount of PCF (r = 0.641, p value <0.001). Graded prolongation of PA-PDI interval was observed across 3 groups of patients divided according to the tertile values of PCF. The AUC for the PA-PDI interval in predicting an increased amount of PCF (third tertile) was 0.796. At a cutoff value of 130 ms identified by the ROC curve, the sensitivity and specificity of PA-PDI interval in identifying patients with a highest tertile of PCF were 63.4% and 85.3%, respectively.

**Conclusions:**

The PA-PDI intervals were longer in patients with an increased amount of PCF. It may be a useful parameter to represent the degree of PCF-related atrial remodeling.

## Introduction

The pericardial fat (PCF), a unique fat deposit due to its proximity to cardiac structures, has been reported to serve as an abundant source of inflammatory mediators which directly damage the heart. [1] An increased amount of PCF has been linked with the presences of coronary calcification and coronary artery disease. [2], [3] Besides, an increasing volume of literature has showed that PCF is significantly increased in patients with atrial fibrillation (AF). [4]–[6] The previous study suggested that PCF may induce local inflammation and subsequent structural remodeling of the left atrium (LA), which potentially contributes to AF genesis or perpetuation. [6] However, the adverse effects of PCF on LA are difficult to be evaluated and quantified clinically.

LA volume measured by transthoracic echocardiogram (TTE) was a non-invasive parameter which was commonly used to evaluate the structural change of LA. However, the measurements of LA volume are often limited by poor temporal and spatial resolution. Recently, a newly developed atrial electromechanical interval on transthoracic echocardiogram (TTE) was reported to be a useful parameter to estimate the total atrial conduction time. [7] Chao et al. further demonstrated that it can reflect the processes of both electrical (decreased voltage, prolonged total activation time) and structural remodeling (chamber enlargement) of LA. [8] In a previous study which enrolled 103 AF and 194 non-AF patients, atrial electromechanical interval, not LA size, is able to identify subjects with AF among those having a small LA. [9] Therefore, we hypothesized that the electromechanical interval may be longer in patients with an increased volume of PCF. It may serve as a convenient tool, which could be more sensitive than LA volume, in reflecting the extent of LA injury due to the adverse effects of PCF. The goal of the present study was to investigate the association between the atrial electromechanical interval and PCF.

## Methods

### Study Population

In this retrospective study, a total of 337 subjects who received cardiovascular health examinations from 2006 to 2008 at the health examination center of Mackay Memorial Hospital in Taipei, Taiwan were included. In addition to blood tests, the health examination project included detailed cardiac examinations, including TTE and multi-detector computed tomography (MDCT). All subjects did not have overt cardiovascular diseases and received these examinations to evaluate their cardiac functions and detect any possibility of significant coronary diseases for screening purposes. All patients did not have the past history of AF, atrial flutter or atrial tachycardia. Subjects were excluded if they had left ventricular ejection fraction <50%, significant valvular heart disease (regurgitation or stenosis), more severe pulmonary hypertension (defined as systolic pulmonary arterial pressure higher than 60 mmHg), chronic lung diseases, existence of congenital heart diseases on echocardiography, known prior cardiac surgery history, and rheumatic heart disease. In addition, there was no previous implantation of pacemaker, overt renal insufficiency (creatinine >2.5 mg/dl), acute or symptomatic coronary events in our study subjects. Data of blood tests, TTE and MDCT were analyzed retrospectively in the present study.

### Lab Data Acquisition

The blood samples were collected and examined for each patient in the fasting state. Lipid profiles and fasting glucose were obtained by a Hitachi 7170 automatic analyzer (Hitachi Corp. Hitachinaka, Ibaraki, Japan). High sensitivity - CRP (hs-CRP) levels were determined using a highly sensitive, latex particle-enhanced immunoassay (Elecsys 2010; Roche Diagnostics GmbH, Mannheim, Germany).

### The Echocardiogram and Atrial Electromechanical Interval

**Figure 1:**
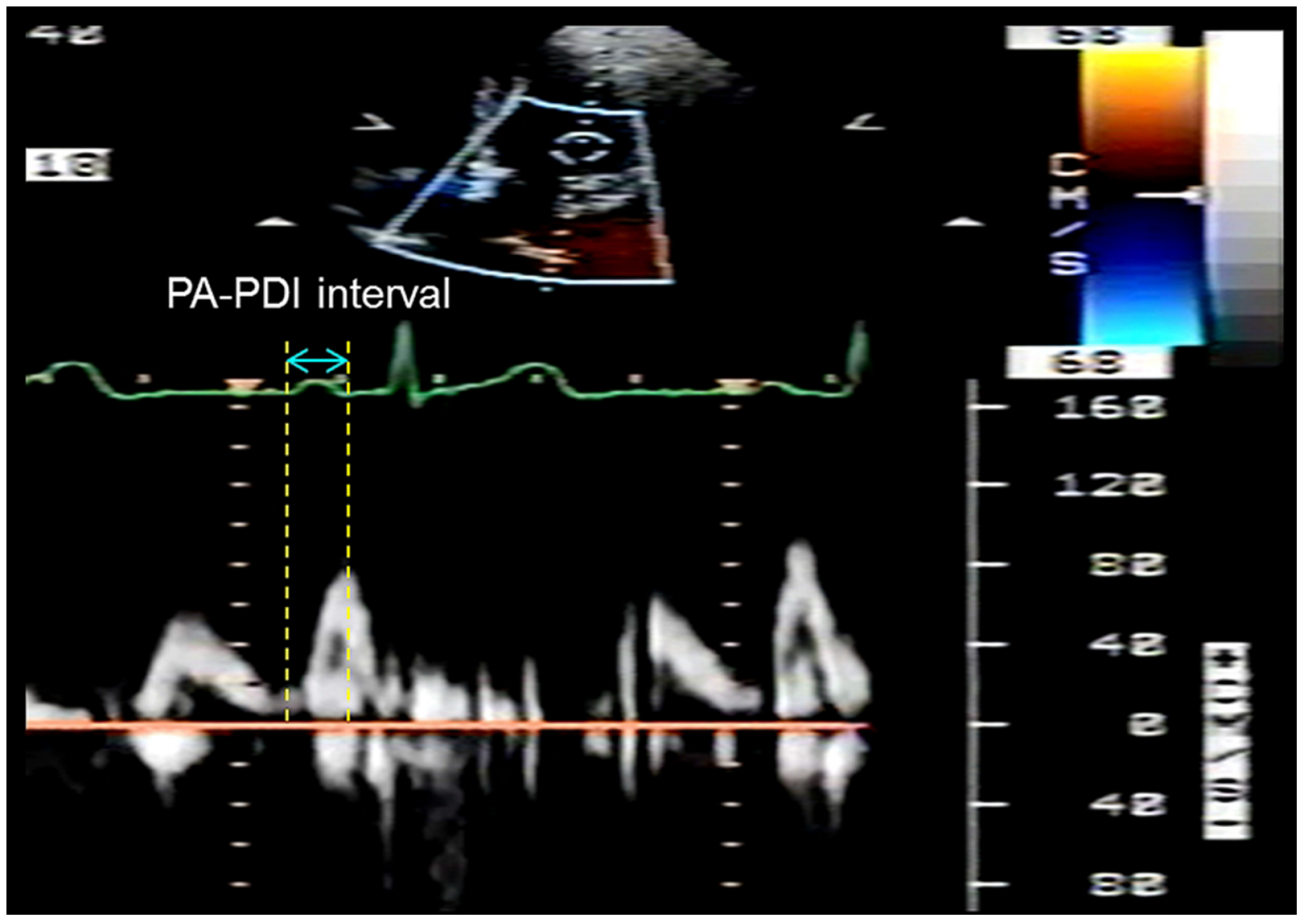
An example of the atrium electromechanical interval (PA-PDI interval) measurement. In the apical four chamber view, the pulse-wave Doppler sample was placed on the mitral inflow tract. The PA-PDI interval was defined as the time-interval from the initiation of the P wave from the electrocardiographic signal (lead II) provided by the echocardiographic machine to the peak of the mitral inflow A wave of the pulse wave Doppler imaging.

TTE was performed using a multi-frequency transducer incorporated in a SONOS 5500 Echocardiograph (Hewlett Packard, Inc., Agilent Technologies, Andover, MA, USA) according to the standard techniques. [10] LA dimension was measured in parasternal long axis view at end systole. The LA volume was measured with a modified biplane area–length method with the echocardiographic tracings made in the apical two- and four-chamber views at end systole. [11] Left ventricular ejection fraction was assessed by Simpson’s method. The atrium electromechanical interval (PA-PDI interval), defined as the time-interval from the initiation of the P wave from the electrocardiographic (ECG) signal (lead II) provided by the echocardiographic machine to the peak of the mitral inflow A wave of the pulse wave Doppler imaging in the apical four-chamber view (Figure 1), was measured in three consecutive cardiac cycles and averaged. The reproducibility of the measurement of the PA-PDI interval has been validated in our previous publication. [8].

### MDCT Scanning Protocol and Measurements of Pericardial Fat

Scanning was performed using a 16-slice MDCT scanner (Sensation 16, Siemens Medical Solutions, Forchheim, Germany) with 16 mm × 0.75 mm collimation, rotation time 420 ms and tube voltage of 120 kV. In one breath-hold, images were acquired from above the level of tracheal bifurcation to below the base of heart using prospectively ECG triggering with the centre of the acquisition at 70% of the R-R interval. From the raw data, the images were reconstructed with standard kernel in 3 mm thick axial, non-overlapping slices and 25 cm field of view.

**Figure 2:**
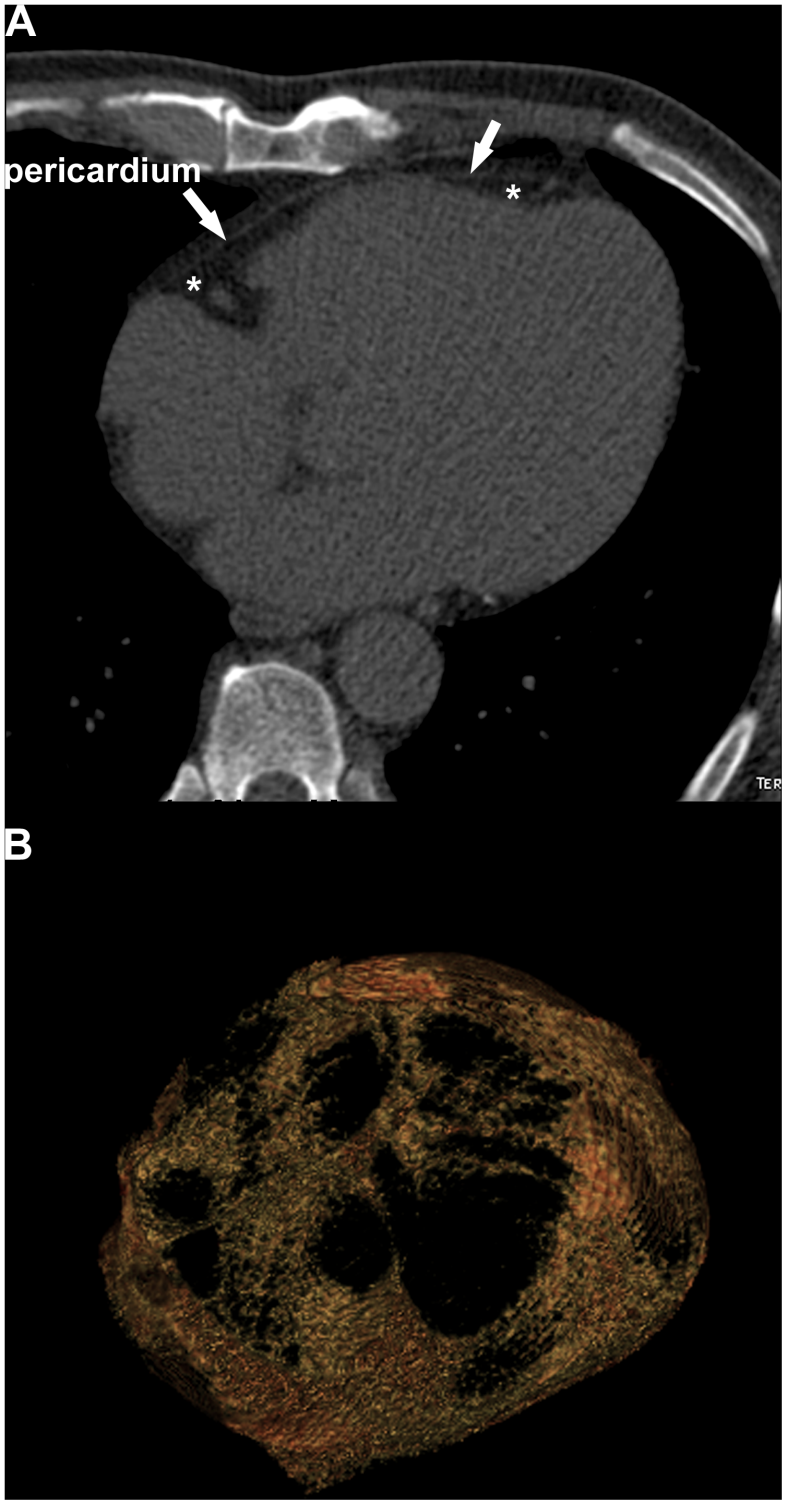
Measurement of pericardial adipose tissue. The PCF (*) was defined by the fat between the heart and the pericardium (arrow) as shown in axial view (A). Thick-slice (10 mm) 3D reconstruction axial view (B) demonstrated PCF. All pixels within a window of −195 to −45 HU and a window centre of −120 HU inside pericardial sac have been selected as PCF and reconstructed into the 3D image. PCF = pericardial fat.

PCF was quantified by MDCT using a dedicated workstation (Aquarius 3D Workstation, TeraRecon, San Mateo, CA, USA). The semi-automatic segmentation technique was developed for quantification of fat volumes. We traced pericardium in axial MDCT images manually from the level of left main coronary artery to diaphragm every four to six slices. The computer software then automatically interpolated and traced pericardium along the manually traced areas. All automatically traced slices were verified and modified if necessary for accuracy. The definition of fat tissue is pixels within a window of −195 to −45 HU and a window centre of −120 HU. PCF was defined as any adipose tissue located within the pericardial sac (Figure 2A and B). The reproducibility of the measurement of the PCF has been validated in our previous publication. [12].

This study complies with the Declaration of Helsinki, and the study protocol was approved by the Institutional Review Board at Mackay Memorial Hospital, Taipei, Taiwan. Because the present study was a retrospective one, the Institutional Review Board agreed that the study can be performed without obtaining the informed consents of study subjects.

### Statistical Analysis

The data are presented as mean values and standard deviations for normally distributed continuous variables, and proportions for categorical variables. The differences between normally distributed continuous values are assessed using an unpaired 2-tailed t test or one-way analysis of variance (ANOVA) with Post Hoc Bonferroni test. The differences between nominal variables are compared using Chi-square test. The association of PA-PDI interval and PCF was analyzed with linear regression analysis. All statistical significances were set at p<0.05 and all statistical analyses were carried out by SPSS 17.0 (SPSS Inc. USA). The statistical powers of the analyses were higher than 80% based on the sample size and a two-sided alpha level of 0.05.

## Results

### Clinical Characteristics of the Study Population

The clinical characteristics of the study population were shown in Table 1. The mean PA-PDI interval was 125.3±14.0 ms with a range from 86 to 163 ms. The mean PCF was 80.6±26.4 mL with a range from 25.2 to 174.8 mL.

**Table 1:** Clinical characteristics of the study population.

Variables	Study population, n = 337
Age, years	51.9±9.0
Male	68.5%
Body mass index, kg/m^2^	24.6±3.0
Waist circumference, cm	84.4±9.0
Medical history	
Hypertension	27.6%
Diabetes mellitus	14.8%
Dyslipidemia	11.9%
Laboratory examinations	
WBCs count, cells/mm^3^	4043±1813
Fasting glucose, mg/dL	100.1±20.0
Cholesterol, mg/dL	199.0±36.5
Triglyceride, mg/dL	142.0±83.4
hs-CRP, mg/dL	0.14±0.15
Transthoracic echocardiogram	
LV wall thickness, mm	9.6±1.2
LVIDD, mm	47.1±3.6
LVEF, %	68.7±3.3
Left atrial diameter, mm	31.3±4.3
Left atrial volume, mL	36.5±12.5
Peak E-wave velocity, cm/s	62.9±13.7
Peak A-wave velocity, cm/s	61.2±15.4
E/A ratio	1.02±0.33
PA-PDI interval, ms	125.3±14.0
Pericardial fat, mL	80.6±26.4

hsCRP = high sensitivity C-reactive protein; LV = left ventricle; LVEF = left ventricular ejection fraction; LVIDD = left ventricular internal dimension at end-diastole; WBCs = white blood cells; PA-PDI = the time interval from the initiation of the P wave deflection to the peak of the mitral inflow A wave on the pulse wave Doppler imaging.

### PA-PDI Interval and Pericardial Fat

**Figure 3:**
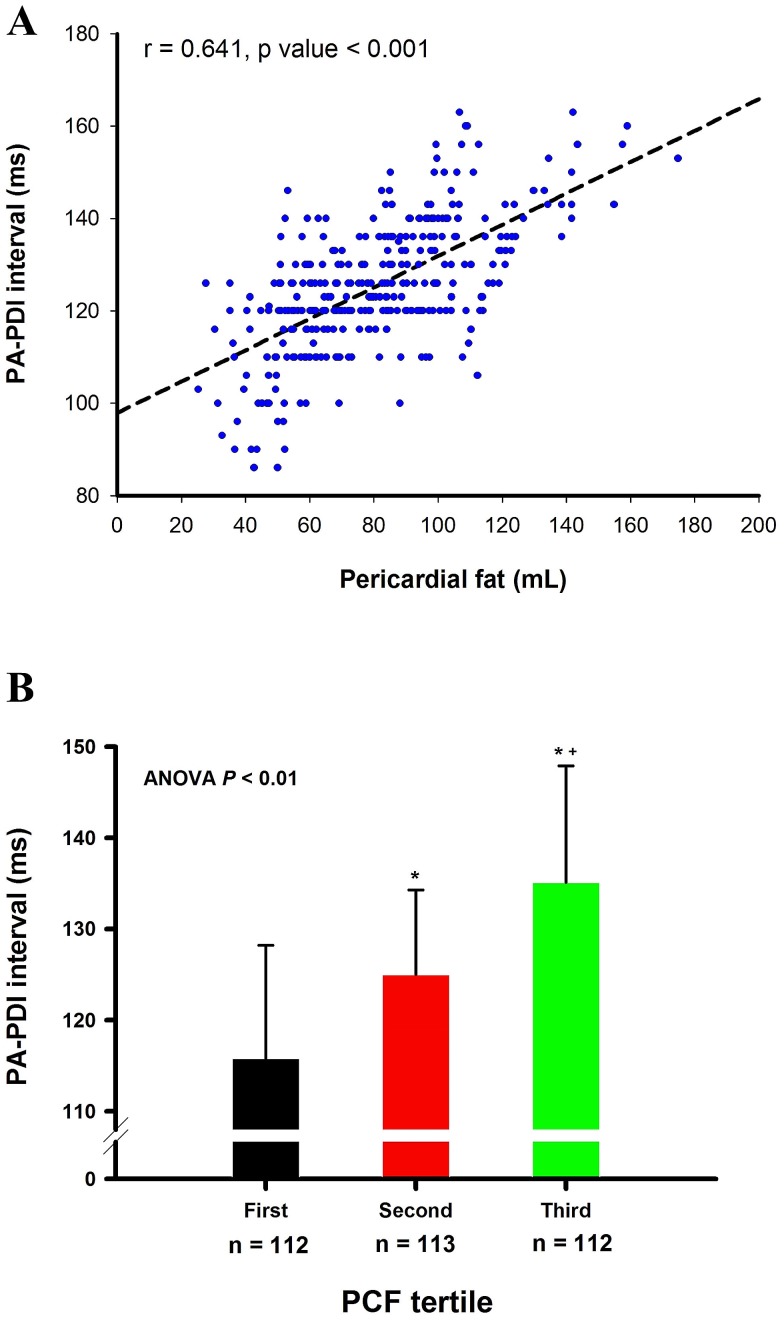
The association between PA-PDI interval and pericardial fat. The PA-PDI interval was significantly correlated with the amount of PCF (r = 0.641, p value <0.001) (A). When patients were divided into 3 groups according to the tertile values of PCF, the PA-PDI interval continuously lengthened from first (115.8±12.4 ms), second (125.0±9.3 ms) to third tertile (135.1±12.8 mm) (B). *P value <0.05, second or third tertile versus first tertile; ^+^P value <0.05, third versus second tertile. PCF = pericardial fat.

The PA-PDI interval was significantly correlated with the amount of PCF (Figure 3A). When patients were divided into 3 groups according to the tertile values of PCF, the PA-PDI intervals continuously lengthened from first, second to third tertile (Figure 3B). The linear correlation between PA-PDI interval and PCF remained significant in different regression models adjusted for multiple variables, including age, gender, BMI, waist circumference, underlying diseases, biochemical data and echocardiographic parameters (Table 2). The results of these models demonstrated that the PA-PDI interval was significantly associated with PCF after taking obesity (model 2), comorbidities (model 3), systemic inflammation (model 4), left ventricular functions (model 5) and LA volume (model 6) into considerations.

**Table 2:** Linear regression models on the relationship between PA-PDI interval and pericardial fat.

Models	β±SE	P value
Model 1: adjusted for age and gender	0.672±0.082	<0.001
Model 2: adjusted for age, gender, body mass index and waist circumference	0.647±0.084	<0.001
Model 3: adjusted for all variables in model 2, and diabetes mellitus, hypertension and dyslipidemia	0.647±0.085	<0.001
Model 4: adjusted for all variables in model 3, and WBCs count, cholesterol, triglyceride, fasting glucose and hs-CRP	0.646±0.088	<0.001
Model 5: adjusted for all variables in model 4, and LV wall thickness, LVIDD, LVEF, left atrial diameter and E/A ratio	0.625±0.091	<0.001
Model 6: adjusted for all variables in model 4, and LV wall thickness, LVIDD, LVEF, left atrial volume and E/A ratio	0.585±0.089	<0.001

hs-CRP = high sensitivity C-reactive protein; LV = left ventricle; LVEF = left ventricular ejection fraction; LVIDD = left ventricular internal dimension at end-diastole; SE = standard error; WBCs = white blood cells.

**Figure 4:**
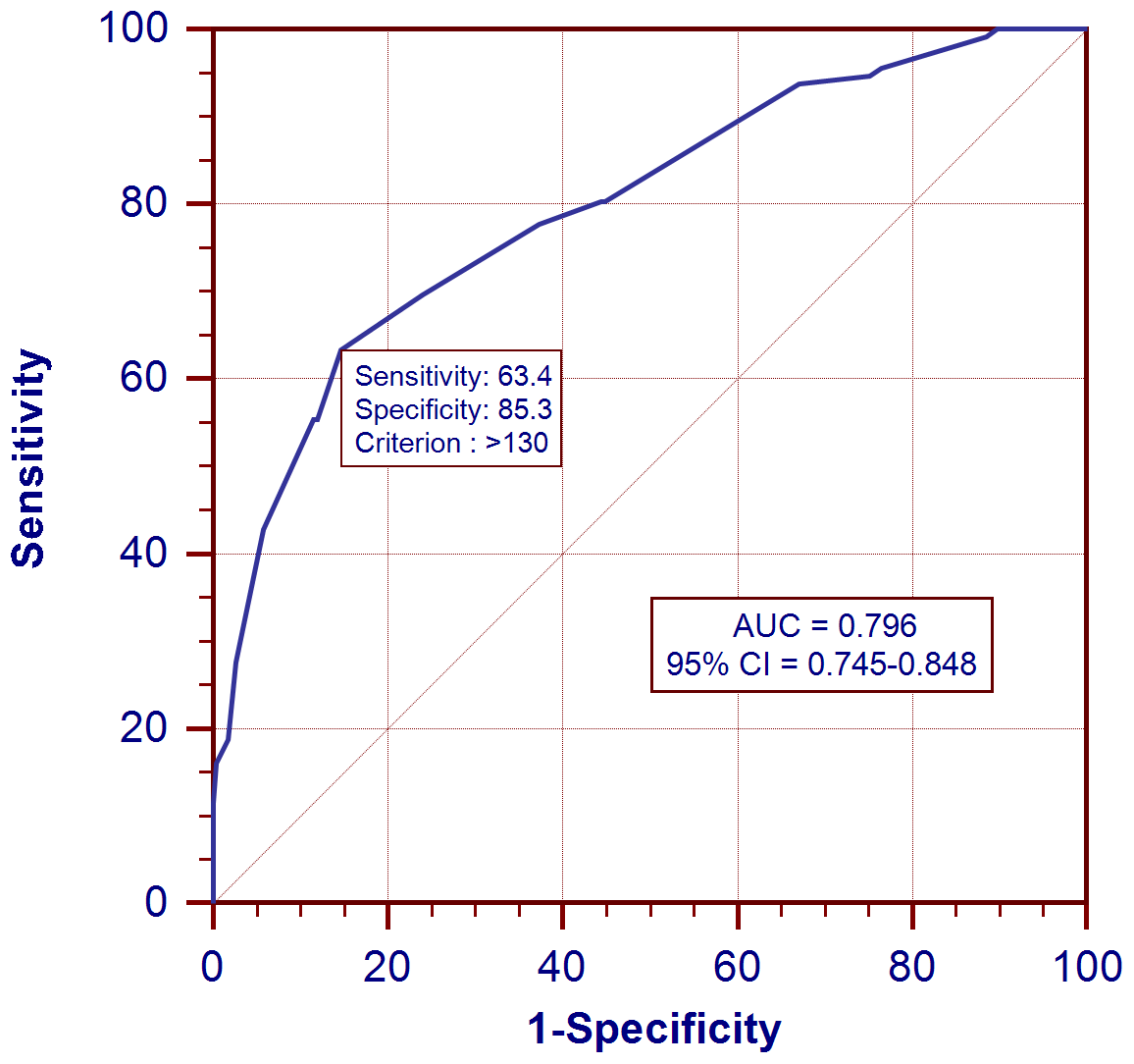
ROC curve for PA-PDI interval in predicting an increased amount of pericardial fat (third tertile). The AUC for the PA-PDI interval in predicting an increased amount of PCF (third tertile, ≥91.9 mL) was 0.796 (95% CI = 0.745–0.848, p value <0.001). At a cutoff value of 130 ms identified by the ROC curve, the sensitivity and specificity of PA-PDI interval in identifying patients with a highest tertile amount of PCF were 63.4% and 85.3%, respectively. AUC = area under the curve; CI = confidence interval; PCF = pericardial fat; ROC = receiver operating characteristic.

The PA-PDI interval was useful in identifying patients with an increased amount of PCF (third tertile, ≥91.9 mL) with an odds ratio of 1.110 per 1 ms increment (95% confidence interval = 1.082–1.139, p value <0.001). The AUC for the PA-PDI interval in predicting an increased amount of PCF (third tertile) was 0.796 (95% CI = 0.745–0.848, p value <0.001)(Figure 4). At a cutoff value of 130 ms identified by the ROC curve, the sensitivity and specificity of PA-PDI interval in identifying patients with a highest tertile amount of PCF were 63.4% and 85.3%, respectively.

### LA Volume and Pericardial Fat

**Figure 5:**
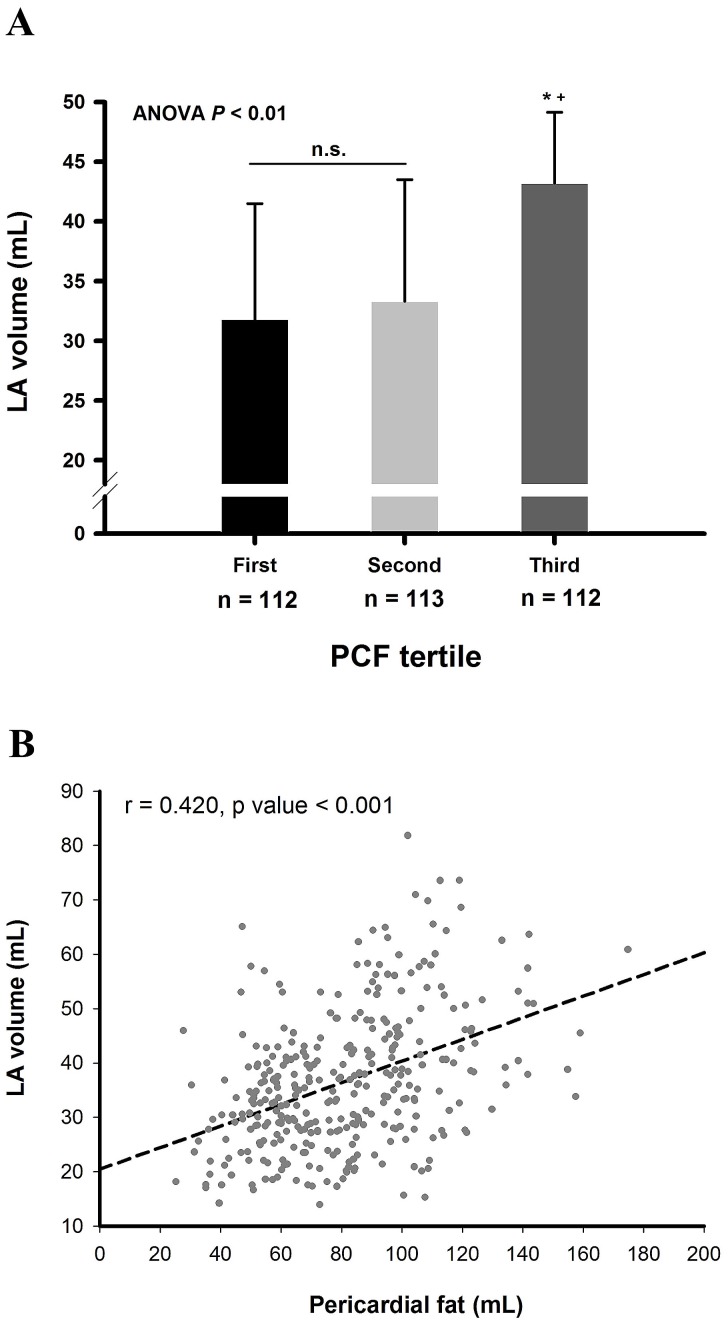
The association between LA volume and pericardial fat. Patients were divided into 3 groups according to the tertile values of PCF, and the LA volume was larger in third tertile compared to that of first or second tertile. However, the LA volume was not different between first and second tertiles (31.2±9.7 mL versus 33.3±10.2 mL, p value = 0.310) (A). LA volume was significantly correlated with the amount of PCF (r = 0.420, p value <0.001) (B). *P value <0.05, third tertile versus first tertile; ^+^P value <0.05, third versus second tertile; N.S. denotes non-significant p value. LA = left atrium; PCF = pericardial fat.

We further compared the volume of LA in different groups of patients divided according to the tertile values of PCF. Similar to the PA-PDI interval, the LA volume was larger in third tertile compared to that of first or second tertile. However, the LA volume was not significantly different between first and second tertiles **(** Figure 5A **)**. Linear regression analysis also showed significant correlation between LA volume and the amount of PCF (Figure 5B).

## Discussion

### Main Findings

In this study, we investigated the associations between the atrial electromechanical interval (PA-PDI interval) and PCF in 337 patients. The main findings were as follows: (1) The PA-PDI interval was positively correlated with the amount of PCF, and may be a useful parameter to quantify the degree of PCF-related LA remodeling. (2) The PA-PDI interval may serve as a more sensitive and convenient tool than LA volume to detect the PCF-related LA damages.

### PCF, PA-PDI Interval and LA Remodeling

PCF represents a unique fat deposit because of its proximity to cardiac structures and its shared blood supply with the cardiac microcirculation. [13] It has been shown to have local inflammatory effects. [1], [14] Tomasz et al. demonstrated that PCF exhibited significantly higher levels of several inflammatory cytokines (interleukin-1, interleukin-6, and TNF-alpha) than subcutaneous fat. [1] Besides, PCF has a threefold higher amount of the proinflammatory cytokine resistin, and fivefold lower amounts of the anti-inflammatory cytokine adiponectin when compared to gluteal adipose tissue. [14], [15] In the study performed by Lin et al. which enrolled a total of 137 AF patients, systemic inflammation, represented by a higher level of hs-CRP, was associated with an abnormal and arrhythmogenic LA substrate. [16] Therefore, PCF serving as an abundant source of inflammatory mediators may increase the local inflammatory burden which directly damaged the LA. The hypothesis was supported by the observation that an increased amount of PCF was associated with a larger LA size, the presence of AF and a higher recurrence rate after AF ablation.[5], [17]–[19].

However, the problem is how to quantify the adverse effects of PCF on LA easily and conveniently. The atrial electromechanical interval, PA-PDI interval, was reported to be able to reflect the degree of structural and electrical remodeling of LA, such as a LA enlargement, prolonged activation time and decreased voltage. [8] The prolongation of the interval was associated with new-onset AF and a higher recurrence rate after AF ablation. [8], [20] In the present study, we demonstrated that the PA-PDI interval was longer in patients with an increased amount of PCF. It was significantly correlated with PCF volume even after adjusting for multiple factors. It may suggest that PA-PDI interval can be used to quantify the adverse effects of PCF on LA independently from obesity, systemic inflammation, left ventricular structures/functions and LA size. At a cutoff value of 130 ms, the PA-PDI interval is helpful in identifying patients with a large amount of PCF (high tertile) with an odds ratio of 7.625 (95% confidence interval = 4.381–12.047). It may serve as a convenient parameter in evaluating the degree of PCF-related LA remodeling.

### Comparisons of PA-PDI Interval and LA Volume in Evaluating PCF-related LA Remodeling

LA volume is a common parameter used in evaluating the process of LA remodeling. Compared with the LA volume calculation, the measurement of the PA-PDI interval was easier because manually traced two-dimensional echocardiographic LA areas are often limited by poor temporal and spatial resolution. The PA-PDI interval could be a alternative choice when precise measurement of the LA volume was difficult. In the present study, LA volume was not significantly different in patients with less amount of PCF (first and second tertiles), which may suggest that LA volume was not sensitive enough to detect the subtle changes of PCF-related LA remodeling. On the contrary, PA-PDI interval continuously lengthened when patients’ PCF volume increased, and was still significantly different between patients with less amount of PCF (first and second tertiles). Besides, the PCF was correlated with PA-PDI interval (r = 0.641) more closely than with LA volume (r = 0.420). These findings suggested that PA-PDI interval, compared to LA volume, may be a better parameter to represent how severe the LA is affected by PCF. Since the subjects enrolled in the present study were those without AF and with a near normal LA size, the prolongation of PA-PDI interval in patients with an increased amount of PCF may imply that the LA has already been damaged in the pre-clinical stage without cardiac arrhythmias, and the PA-PDI interval may be sensitive enough to detect these subtle changes before the structural remodeling happened.

### Limitations

There were several limitations of the present study. First, we analyzed the relationship between PA-PDI interval and the total amount, rather than regional distribution of PCF. Therefore, we were not able to investigate whether a specific site of PCF played a more important role in LA remodeling than other sites. Second, some precise echocardiographic parameters of LA, such as LA strain and strain rate, were not available in the present study. However, the purpose of our study was to provide a convenient and easy-measured clinical parameter with potentials in quantifying the degree of PCF-related atrial remodeling, rather than claiming that the PA-PDI interval was better than other echocardiographic tools. Third, the study subjects were patients who received cardiovascular health examinations at the medical center, and therefore a selection bias may exist. Lastly, since patients with atrial arrhythmias and overt cardiovascular diseases were not enrolled in the present study, whether the results presented here can be applied to patients with atrial arrhythmias or significant cardiac diseases remained uncertain.

## Conclusion

The PA-PDI intervals were longer in patients with an increased amount of PCF. Compared to LA volume, the PA-PDI interval may be a more sensitive parameter to represent the degree of PCF-related atrial remodeling.
